# News and Updates from 2022 on Antioxidant and Anti-Inflammatory Properties of Marine Products

**DOI:** 10.3390/md21010026

**Published:** 2022-12-29

**Authors:** Marzia Vasarri, Donatella Degl’Innocenti

**Affiliations:** Department of Experimental and Clinical Biomedical Sciences, University of Florence, Viale Morgagni 50, 50134 Florence, Italy

Inflammation and oxidative stress are often the common denominators of most modern chronic diseases and disorders, resulting in serious problems for health care systems. The problems associated with the long-term use of conventional therapies have prompted research into new safe, effective, and natural anti-inflammatory and antioxidant agents. In this context, the scientific community is focusing its attention on the rich biodiversity of the marine environment as a molecular source of a variety of bioactive compounds.

This Special Issue of *Marine Drugs*, “Marine Anti-inflammatory and Antioxidant Agents 2.0”, brings together six original research papers, two clinical studies, and two comprehensive reviews of natural marine anti-inflammatory and/or antioxidant agents. The natural compounds discussed were sourced from a variety of marine organisms, including oysters, seaweeds, marine plants, deep-sea fungi, Chondrichthyes, and echinoderms. The collected scientific works introduce new perspectives on the applicability of marine products for human health and in biotechnology. 

Among all marine organisms, seaweeds have been used since ancient times for their many health benefits, which are mainly due to their bioactive compounds. Therefore, research on the biological properties of seaweed-derived compounds has made considerable progress in various fields of applications possible. In this regard, El-Beltagi et al. (2022) collected information on the composition and beneficial properties of seaweeds [1]. This review described seaweeds as an alternative source of synthetic substances to improve consumer well-being through their incorporation into novel foods or functional medicines.

Research by Roach et al. (2022) also demonstrated valuable health and pharmacological properties of seaweed-derived compounds [2]. Two clinical studies were conducted in humans after ingestion of a single sulfated polysaccharide from the green alga *Ulva* sp. 84, “xylorhamnoglucuronan” (SXRG84). Study 1 found a reduction in non-HDL (high-density lipoprotein) cholesterol and the atherogenic index in all participants, in the insulin levels in overweight adult participants fed 2 g/day of SXRG84, and in the C-reactive protein levels in overweight participants fed 4 g/day of SXRG84. Study 2 described an unchanged lipid level between the groups and lowered plasma concentrations of pro-inflammatory cytokines (IFN-γ, IL-1β, TNF-α, and IL-10) at 12 weeks after SXRG84 treatment. The two studies did not show consistent effects on the gut microbiome, although a change in microbiota composition and abundance following treatment was observed in Study 1. Given the beneficial effects of SXRG84 on inflammatory markers in overweight subjects, the authors suggested the potential use of SXRG84-based supplements to reduce the inflammatory response related to metabolic disorders.

Beside seaweeds, many other marine organisms are sources of polysaccharides. Chondroitin sulfate (CS), an anionic glycosaminoglycan derived from animal cartilage, is widely used in various biopharmaceutical applications. This Special Issue presents a theoretical study on the use of CS derived from shark cartilage as a stabilizing agent of selenium nanoparticles (SeNPs) [3]. Selenium (Se) is an essential micronutrient for human health due to its antioxidant properties, but the narrow margin between beneficial and harmful doses limits its practices in food and medicine. Furthermore, the advantages of using SeNPs as drug carriers are limited by stability issues. Chen et al. (2022) prepared selenium-chondroitin sulfate (SeCS) via a redox reaction of sodium selenite and ascorbic acid, using shark CS as a template. SeCS possessed good storage stability and had higher antioxidant activity than SeNPs and CS [3]. Therefore, the authors proposed the application of SeCS in the prevention and mitigation of oxidative stress-related diseases.

Marine bioactive natural products also include peptides and proteins. The broad spectrum of the bioactivity of these biomolecules makes them potentially valuable nutraceuticals and medicines.

A huge body of literature indicates that the human health benefits of protein hydrolysates can, in part, be attributed to their free-amino-acid-rich and peptide-rich properties. Wang et al. (2022) described how free-amino-acid-rich protein hydrolysates obtained from oysters (*Crassostrea hongkongensis*) had an ameliorative effect of on cadmium (Cd)-induced acute liver injury in mice [4]. As a result of the treatment with oyster protein hydrolysates (OPs), liver function profiles were improved, whereas hemorrhages, lymphocyte accumulation, and inflammatory cell infiltration around the central veins were reduced. Supplementation with OPs reduced malondialdehyde formation and restored the activity of antioxidant enzymes (SOD, CAT, and GPH-Px) in the liver of Cd-exposed mice. OPs blocked inflammatory responses (IL-1β, IL-6, and TNF-α) by inhibiting the expression of inflammation-related proteins (MIP-2 and COX-2) and suppressed hepatocyte apoptosis (Bax, caspase-3, and Blc-2) by regulating ERK/NF-κB- and PI3K/AKT-related signaling pathways in Cd-exposed mice. 

Among marine organisms, microalgae represent another rich source of proteins and peptides. *Chlorella vulgaris* is a green microalga used as a source of proteins in aquatic feed to improve growth performance, oxidative status, and immune response in several fish species. The study by Reis et al. (2022) evaluated the effects of short-term supplementation with a 2% *C. vulgaris* biomass and two 0.1% *C. vulgaris* soluble peptide-enriched extracts on immune defenses, oxidative stress, and the inflammatory response of gilthead seabream (*Sparus aurata*) after an inflammatory insult [5]. The *C. vulgaris* peptide-enriched extract had a dual modulatory effect at both the blood and gut levels. In particular, the peptide-enriched extract drives the proliferation of circulating neutrophils in resting gilthead seabream, and, following an inflammatory insult, it can protect the gut from stress.

Carotenoids are among the most common pigments found in the marine environment, and commercially appealing applications are possible due to their many biological properties. Astaxanthin (ATX) is a lipid-soluble carotenoid found in most marine organisms that has documented pharmacological effects, including neuroprotection and antioxidant activity. The study published by Park et al. in 2022 found that the once-daily administration of ATX (100 mg/kg) significantly reduced the death of hippocampal pyramidal cells in gerbils undergoing transient ischemia, as well as DNA damage and lipid peroxidation in the forebrain pyramidal cells [6]. Furthermore, ATX treatment increased the expression of the antioxidant enzyme superoxide dismutase. Therefore, the authors suggested that ATX, due to its antioxidant and neuroprotective properties, can be used as a potential dietary supplement to prevent the progression of severe ischemic brain injury.

The marine environment is also an excellent source of phenolic compounds. Marine-derived phenols exhibit a wide range of biological effects through their uniqueness and structural complexity. The study by Morresi et al. (2022) showed that polyphenol-rich *Posidonia oceanica* leaf hydroalcoholic extract (POE) can reduce glucose transport by lowering GLUT2 levels, promote intestinal barrier integrity by modulating levels of Zonulin-1, and have a protective antioxidant effect against glucose-induced damage in human intestinal Caco-2 cells [7]. This was the first study investigating the behavior of a complex pool of POE phenolic compounds on differentiated Caco-2 cells as a model of the intestinal barrier. Therefore, the authors suggested that the phytocomplex of *Posidonia oceanica* may prevent gut cell dysfunction during the development of inflammation-related diseases associated with oxidative stress.

Furthermore, Vodouhè et al. (2022) demonstrated that the daily consumption of 500 mg of polyphenol-rich brown seaweed (*Ascophyllum nodosum* and *Fucus vesiculosus*) capsules combined with a low-calorie diet had no impact on body weight and blood sugar in overweight prediabetic subjects [8]. Instead, the intake of polyphenol-rich capsules showed a beneficial impact on insulin secretion, heart rate, and inflammatory response. Considering these data, the authors suggested that the early effect of brown seaweed extract on the inflammatory response might be associated with marginal changes in metabolic parameters related to the prevention of type 2 diabetes.

Looking for new bioactive compounds from marine natural products, Anh et al. (2022) isolated and identified three nitrogen-containing secondary metabolites from the ethyl acetate extract of the marine fungus *Aspergillus unguis* IV17-109 [9]. In particular, two new compounds, variotin B (1) and coniosulfide E (2), were identified together with a known compound, unguisin A (3). Compounds 1 and 2 were preliminarily tested for their anti-inflammatory activity by evaluating their inhibitory effect on the lipopolysaccharide-induced production of inflammatory mediators (including NO, IL-6, and iNOS) in RAW 264.7 murine macrophages. Interesting results were obtained for compound 1, which showed moderate anti-inflammatory activity.

In recent decades, many studies have focused on the anti-inflammatory effects of compounds from echinoderms. In this context, Ghelani et al. (2022) collected research articles published between 2010 and 2022 on the anti-inflammatory properties of sea cucumbers, sea urchins, and starfish [10]. In this review, the structures, bioactivity, and molecular mechanisms of these compounds were summarized. In addition, the potential applications of these compounds in the pharmaceutical industry for drug development against chronic inflammation were highlighted.

In conclusion, the studies compiled in this Special Issue confirm that marine organisms are an excellent source of biologically active molecules with antioxidant and anti-inflammatory properties, and that studies of these marine products can contribute to the discovery of new drugs and the scientific validation of their use.
